# Revealing the Underlying Mechanisms in Performance Enhancement of Shellular Lattice Electrodes During Anion Exchange Membrane Water Electrolysis Process

**DOI:** 10.1002/advs.77317

**Published:** 2026-08-21

**Authors:** Tianxiao Niu, Rui Li, Xiao Chen, Deyong Sun, Tianyu Gao, Junhao Ding, Shuo Qu, Xu Song

**Affiliations:** ^1^ Department of Mechanical and Automation Engineering Chinese University of Hong Kong Shatin Hong Kong SAR China; ^2^ School of Materials Science and Engineering North China University of Water Resources and Electric Power Zhengzhou China; ^3^ Department of Engineering Mechanics Zhejiang University Hangzhou Zhejiang China

**Keywords:** anion exchange membrane water electrolysis, computational fluid dynamics simulation, gas diffusion layer, micro laser powder bed fusion, triply periodic minimal surface lattice

## Abstract

Anion exchange membrane water electrolysis (AEMWE) is widely considered as a pragmatic approach for next‐generation hydrogen production. The constraints on its efficiency mostly arise from the traditional gas diffusion layer (GDL), which is made of a random open‐cell structure that traps bubbles within its porous matrix. Utilizing micro laser powder bed fusion 3D printing technology, we designed and fabricated regular open‐cell continuous shellular lattice GDLs that manage the gas‐liquid flow. Precision‐controlled Gyroid lattice achieves advanced‐catalyst‐level overpotentials, a more‐than‐twofold increase in charge‐transfer capacity, and electrochemical stability over 640 h. Despite Fischer–Koch S structure offering the highest real surface area factors, the Gyroid structure outperforms other lattices due to enhanced bubble escape phenomenon and convective replenishment of reactants. Multifactor parametric analysis reveals that mass‐transfer factors dominate over real‐surface‐area factors in performance enhancement, as they can minimize bubble retention and expand active site accessibility, thereby suppressing overpotential. The continuous shell geometry also provides uninterrupted electron pathways and hydrophilic surface control, reducing gas entrapment compared to commercial GDLs. These findings suggest that the architected thin‐walled nickel shellular lattices are superior diffusion layer structure designs for AEMWE, which highlight the synergy of mass‐transfer and surface‐exposure as a general strategy for electrolysis cell component design.

## Graphical abstract

Micro laser powder bed fusion enables architected nickel shellular lattice gas diffusion layers for anion exchange membrane water electrolysis. Among regular open cell geometries, the Gyroid lattice promotes bubble release, convective reactant replenishment and continuous electron transport, delivering low overpotentials, enhanced charge transfer and stable operation over 640 h through mass transfer‐dominated performance gains.

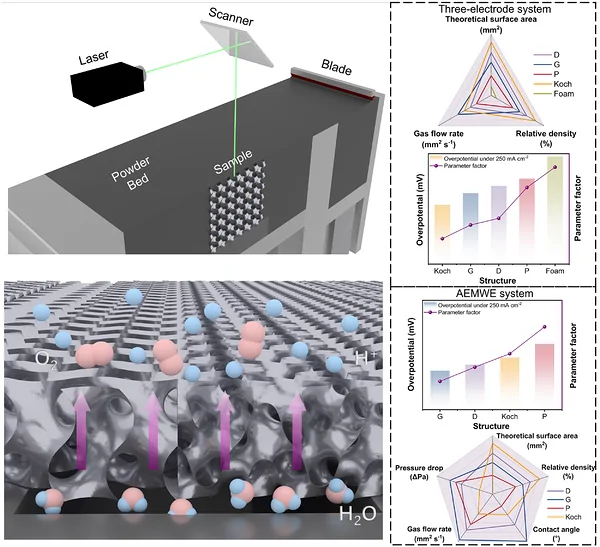

## 1 Introduction

Hydrogen has emerged as an important high‐energy‐density and environmentally friendly alternative to fossil fuels, with its industrial‐scale deployment becoming the key to the global sustainable energy transition [1, 2]. The production of green hydrogen by water electrolysis, utilizing renewable energy sources, has gained significant interest as an attractive technique for the production of clean and sustainable energy [3]. The alkaline water electrolysis (AWE), a conventional water electrolysis technology, is inadequate for mass production due to its elevated ohmic resistance and diminished hydrogen output per system volume [4, 5, 6]. The proton exchange membrane water electrolysis (PEMWE), despite its comparatively superior cell performance, necessitates noble‐metal catalysts and acid‐resistant materials, leading to a much higher equipment cost [7, 8, 9]. Among various electrolysis technologies, the anion exchange membrane water electrolysis (AEMWE) is emerging as a transformative hybrid technology that combines alkaline‐stabilized non‐precious‐metal electrocatalysts with membrane‐mediated ionic conduction to produce high‐purity hydrogen while minimizing system complexity [10, 11, 12].

Gas‐diffusion layers (GDLs) are pivotal yet geometrically underoptimized components in the AEMWE system, which function as the interface between the membrane electrode and the flow channel, simultaneously manage electron conduction, heat dissipation, mechanical support, and gas‐liquid transport [13, 14]. In principle, a well‐architected porous network should admit liquid uniformly, evacuate product gas rapidly, and provide geometrically continuous pathways for charge transport and load‐bearing for the catalyst [15, 16]. However, commercially used titanium fiber felts [17, 18] and nickel foams [19, 20] comprise stochastic fiber/strut networks that trap bubbles within their tortuous pores. Gas retention blocks liquid ingress, disrupts wettability, elevates local ohmic and concentration losses, and ultimately drives higher cell voltages and sluggish kinetics [21, 22]. Beyond gas transport, uncontrolled outer surface roughness and low contact region with the membrane in random media complicate electrolysis cell integration and long‐term mechanical stability [23]. These limitations motivate a paradigm shift from irregular porous foam to geometry‐controlled lattice structure design, which offers a new route to minimize bubble detention, sustain convective replenishment, and stabilize electronic transmission. The design and fabrication of GDLs with a geometrically controllable lattice topology, suitable mechanical and surface properties, as well as low cost, has become increasingly vital.

The LPBF technique is distinguished by its high manufacturing precision, excellent process repeatability, and inherent cost efficiency, making it an attractive route for fabricating advanced electrode architectures [24, 25]. This additive manufacturing method can create complex surfaces that better suit the requirements of new GDLs. The triply periodic minimal surface (TPMS) lattice structures, characterized by a three‐dimensional surface with zero mean curvature, possess a high specific surface area and good geometric continuity, rendering them unique advantages for catalysis and heat exchange applications [26, 27]. Contrary to those in random foams, bubbles can be seamlessly transported along the inner wall of TPMS lattices, facilitating effective gas‐liquid coupled flow [28]. Rocha et al. demonstrated the feasibility of printed Inconel 625 electrodes by integrating high specific surface area with bubble detachment functionality in a TPMS structure created by LPBF technology [29]. Xu et al. explored bubble growth and detachment trajectories in printed nickel TPMS lattice electrodes with a low 1.63 V overpotential at 500 mA cm^−2^ [30]. However, the excessive wall thickness with larger unit cell size at low precision increased the actual thickness of the printed structure, leading to higher flow resistance when the gas‐liquid mixture is discharge [31]. Furthermore, the higher wall thickness poses challenges for finite element modeling in capturing the internal flow distribution, obscuring the mechanism study of the diffusion layers. Consequently, the development of GDLs with TPMS lattice structures characterized by minimal wall thickness and large continuous holes holds great potential for significantly enhancing electrochemical performance.

In this work, we establish an integrated design to architect nickel GDLs that synergize high mass transfer with high surface exposure in AEMWE. Using a proprietary stereolithography (STL)‐free, single‐path micro‐LPBF process, we are able to print thin‐walled TPMS shellular lattices with wall thicknesses below 60 µm for the first time, enabling the synergetic behavior of rapid bubble disengagement while maintaining continuous electronic pathways [32, 33]. A library of shellular lattice geometries was systematically evaluated, both as anode electrodes in a three‐electrode system and as anode GDLs in a full AEMWE system. Among tested TPMS structures, the Fischer‐Koch S lattice structure, characterized by a high surface area, delivered optimal electrochemical performance in the electrolytic cell, whereas the Gyroid structure, noted for its high mass transfer efficiency, outperformed others in the AEMWE. To elucidate such distinctive phenomena, we reconstruct face‐sheet STL‐tied models and implement coupled simulations, bridging micro‐scale gas‐liquid mixing with macro‐scale porous‐medium transport. We propose this dual‐route workflow with STL‐free geometric modeling for fabrication and STL‐tied geometry reconstruction for simulation, which integrates the design, printing, and mechanism study for thin‐walled lattice GDLs and electrodes. We applied a normalized, weighted scoring framework to disentangle the effects associated with the real surface area from those governed by mass transfer, thereby enabling a balanced comparison of different key influencing factors for different 3D‐printed GDL architectures. The findings indicate that, in the AEMWE system, the mass transfer capability of GDLs exerts a greater influence on electrochemical performance than the real surface area.

## 2 Results and Discussions

### 2.1 Structural Characterization of 3D Printed Shellular Lattice GDLs

The Ni GDLs with TPMS structures were fabricated by a dual‐route geometric modelling process, as shown in Figure 1a. The process for constructing lattice structures introduces an innovative approach for printing thin‐walled gas diffusion layers with adjustable, tailored configurations. In detail, the corresponding lattice structures or single‐layer data path mathematical formula is directly converted into the printer's laser toolpath. This STL‐free route methodology omits the traditional modeling processing method of translating the STL file format into a toolpath, which makes it possible to generate structures with small wall thicknesses, while resolving the issue of print failures caused by erroneous planar paths, which occur due to the conversion of STL into toolpaths, hence enhancing the success rate and efficiency of path generation [32]. The printed sample is used for water electrolysis testing. Simultaneously, the corresponding surface of the lattice structure is generated using MATLAB, and the triangular face sheets of the implicit surface are exported in the STL file format. To utilize the mesh in simulation software for solid identification and generation, the surface is created by extracting feature edges, followed by the generation of a simplified reconstructed mesh from the surface. This process yields an STL mesh of sufficient quality to facilitate simulations. Figure 1b displays the operational schematic of micro‐LPBF 3D printing. The translational motion of the recoating blade evenly spreads the powder across the powder bed. The laser is aimed at the substrate via the biaxial movement of the scanner to melt the spread layer of powder. The micro‐LPBF employs as little as 60 W to melt the nickel powder, which is sufficient to create narrower print tracks. This bottom‐up printing approach enables the accurate production of intricate features that are otherwise difficult to achieve by using conventional methods. Given that the curvature radius of the TPMS structure is greater than a critical value, and the angle between the tangent plane and the horizontal plane at any point is constantly greater than 45°, these characteristics can make printing possible with self‐support in micro‐LPBF, which also improves the success rate.

**FIGURE 1:**
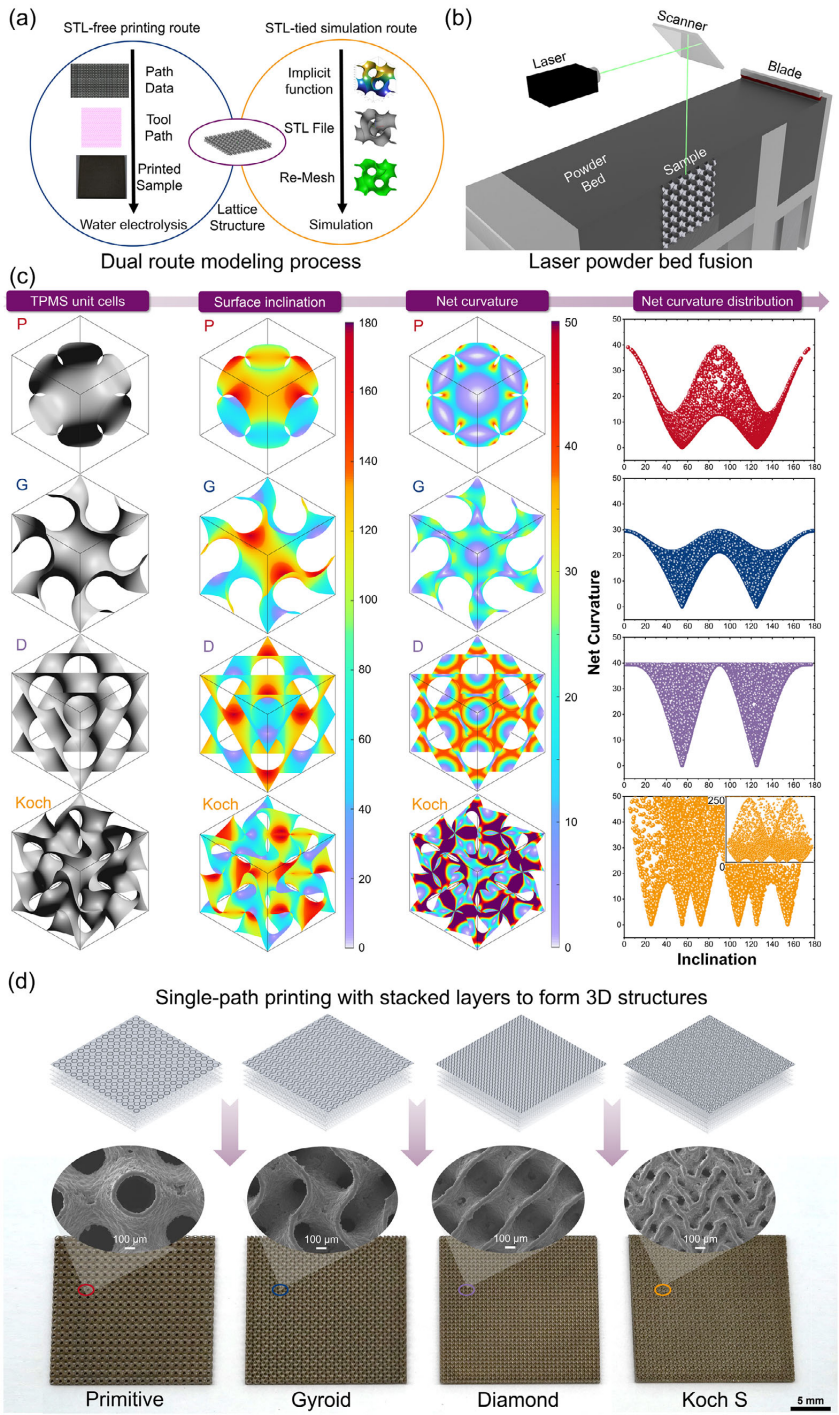
(a) Dual‐route geometric modeling process. (b) Schematic diagram of micro‐LPBF 3D printing technology. (c) Design of four structural types and distribution of inclination and net curvature at each point on the surface. (d) Macroscopic and corresponding microscopic topology of different 1 mm unit‐cell cellular lattice structures.

Figure 1c illustrates four TPMS structures designed using implicit functions: Primitive (P), Gyroid (G), Diamond (D), and Fischer‐Koch S (Koch). The P structure demonstrates the largest pore size, functioning as a simple cubic symmetric configuration marked by a periodic variation in pore size governed by the cosine function, resulting in limited connectivity that facilitates energy storage but complicates fluid application. Conversely, the G structure features a smooth, continuous double helix channel with uniformly distributed pore sizes, high pore connectivity, and low hydrodynamic resistance, particularly in mass transfer and heat exchange applications. The D structure characterizes tetrahedrally symmetric pores with lattice‐level structural gradients and dislocations, resulting in high mechanical strength, primarily utilized for energy absorption applications. Koch fractal geometries feature complex multi‐level porosity, exhibiting enhanced anisotropy and providing the highest specific surface area, making them commonly used in surface heat transfer applications. The correlation between inclination and net curvature in various structures elucidates their topological character. The P structure demonstrates inclinations primarily at 0°, 90°, and 180°, with curvature broadly ranging from 0 to 40°, revealing dispersed flow characteristics along the wall surface. The G structure presents relatively uniform inclinations between 20° and 160°, with curvature concentrated in the 10–30° range, indicating the smoothest curvature variations and continuous bending, reflecting smooth flow characteristics along the wall surface. The D structure shows inclinations near 55° and 125°, with curvature exhibiting a distinct bimodal distribution between 0 and 40°, indicating specialized flow along non‐orthogonal wall directions. The Koch structure displays multi‐peak, multi‐trough high‐frequency fluctuations with non‐strict periodic symmetry. Its curvature reaches 250 with multiple maxima, exhibiting multiple streamlines along the wall and flow separation characteristics. Figure 1d exhibits four 3D‐printed TPMS structures, which are engineered to produce macrostructures layer by layer from single‐path patterns. Together with their respective SEM electron micrographs, organized from left to right are P, G, D, and Koch structures. At the millimeter scale, the surface finishes of the various TPMS structures were adequately refined to satisfy the geometric specifications of the formulations. At the micrometer scale, the overall structural integrity, with cured layer widths maintained within 60 µm for each layer of the single‐path printing, demonstrates the precise printing capability of the micro‐LPBF. The corresponding structure of the 0.5 mm unit cell is shown in Figure S1. Compared with the 1 mm unit cell structures, the 0.5 mm unit cell structures lack boundary continuity. This is because the smaller local curvature cannot be printed continuously, requiring segmented printing, which reduces printing accuracy. Figure S2 demonstrates the SEM images of the G structure as a typical structure at various scales to enhance the investigation of the printed surface morphology. The 3D‐printed Ni surface at the 100 µm scale exhibits a wave‐like concave‐convex structure resulting from rapid cooling, which markedly enlarges the real surface area of the GDL and offers additional contact sites for catalytic reactions. The Ni particles, ranging from 1–10 µm in size, are observed embedded within the interstitial spaces of the molten layer at the scale of 10 µm. The powder is uniformly distributed among the molten layers, while the interstitial spaces between the powder and the molten substrate create micrometer‐sized pores (0.1–5 µm), thereby enhancing the specific surface area. The inter‐micron grain boundaries are distinctly visible, with localized nanoscale etch holes on the surface. Overall, compared to the same TPMS structures with significantly thicker walls, the thin‐walled structure exhibits a larger pore size, thereby facilitating bubble escape once the electrolytic reaction commences in water.

### 2.2 Electrochemical Performance of Electrode in the Three‐Electrode System

The electrochemical performance was evaluated in the three‐electrode system, with the printed structure as the working electrode, a carbon rod as the counter electrode, and Hg/HgO as the reference electrode. Figure 2a presents the linear sweep voltammetry (LSV) curves of various TPMS structures at a projected area corresponding to the current density, which shows the relationship between potential and current density. All the structures exhibit comparable polarization voltages owing to the utilization of pure nickel material. The Koch structure exhibits the most rapid escalation in current density due to the higher density of active sites by its superior specific surface area, followed by the G, D, and P structures, while the Foam structure shows the least change. The Tafel slope plot (Figure 2b) represents the reaction kinetics of the electrode, and a reduced Tafel slope facilitates a more rapid reaction. The G structure exhibits an unexpectedly low Tafel slope of 64.78 mV dec^−1^, succeeded by the D structure at 65.43 mV dec^−1^, Koch at 68.67 mV dec^−1^, and P at 73.15 mV dec^−1^, while Foam presents the highest Tafel slope at 90.23 mV dec^−1^, thereby highlighting the superiority of the 3D‐printed TPMS structures in terms of catalytic effect. For thin‐walled structures, the projection shape is not a purely two‐dimensional square; instead, it is a porous configuration characterized by numerous holes rather than solid forms, exhibiting a tortuous path, as the average area of each layer is below 20% of the two‐dimensional square. Therefore, the average area obtained from cross‐sectional areas spaced at 0.1 mm intervals was used to calculate the current density, and an LSV diagram was plotted (Figure 2c). Normalizing the cross‐sectional area can mitigate the current density variation induced by surface area variables. Although the P structure exhibits excellent current density growth due to its smallest cross‐sectional area, the G structure demonstrates the fastest current density growth rate, showing the overpotential of 348.16 mV under 250 mA cm^−2^. The Koch structure, with its high cross‐sectional area, exhibits the lowest current density growth. Figure S3g shows the LSV plots for five different structures normalized by electrochemically active surface area (ECSA) calculated from double‐layer capacitors (Cdl). Due to the extremely high ECSA of the Koch structure, its overpotential is higher than that of the Foam structure after normalization, while the G structure has the lowest overpotential due to its high performance at lower ECSA. This indicates that the low overpotential of the Koch structure in the three‐electrode system is contributed by its surface area factor. Figure S4 shows the LSV plots of six printed G‐structure samples, and the consistent LSV curves demonstrate that low manufacturing variability results in minimal performance differences, confirming the operational repeatability of thin‐walled shell lattice micro LPBF manufacturing. Corresponding testing of the 0.5 mm unit cell structures (Figures S5 and S6) demonstrated similar performance regardless of structure, despite their increased surface area. This phenomenon occurs because, once the reaction initiates, bubble formation obstructs the small pores of the 0.5 mm unit cell, thereby considerably diminishing the effective reaction area. This suggests that employing excessively small pore sizes to increase surface area is inappropriate in electrolytic water processes. Moreover, all TPMS structures showed an increase in resistance relative to commercial Foam, making them suitable alternatives to commercial nickel foam. Stability testing of the G structure sample in a three‐electrode system was conducted for over 1500 h (Figure 2d), The test was conducted at a current density of 500 mA cm^−2^, with the potential relative to RHE starting from 1.986 V. The potential gradually decreased to around 1.96 V in the first 367 h, which is attributed to the dynamic surface Ni(OH)_2_/NiOOH reconstruction of the printed structure, the formation of hydroxyl oxide active phase, and the increase in benign surface area through electrochemical re‐deposition of surface nano nickel. Subsequently, the voltage stabilized for 1189 h, fluctuating between 2.01 V and 2.04 V, and exhibited no substantial failure at its final stage, so illustrating the consistency of its performance. Figure S7a shows the microstructure after the stability test. The overall microscale structure has a full shape without any collapse or loss of the overall geometric structure. Ni(OH)_2_/NiOOH reaction structure protrusions were developed on the nanoscale surface, indicating a stable structural advantage. Figure S8 illustrates the location of the oxidation‐reduction peak of the G structure, positioned at 1.439 V vs. RHE, signifying that the surface is capable of facilitating the transformation of Ni(OH)_2_ to NiOOH during stability assessments. Figure S9 depicts the Raman spectra prior to and after the reaction, featuring two distinctive peaks corresponding to β—NiOOH and γ—NiOOH at 473 cm^−1^ and 548 cm^−1^, respectively, signifying surface reconstruction. Figure S10 shows the comparison of LSV before and after stability. The oxidation peak around 1.35 V is more pronounced after the reaction, and the electrochemical performance is better than at the initial stage, reflecting the effect of surface reconstruction.

**FIGURE 2:**
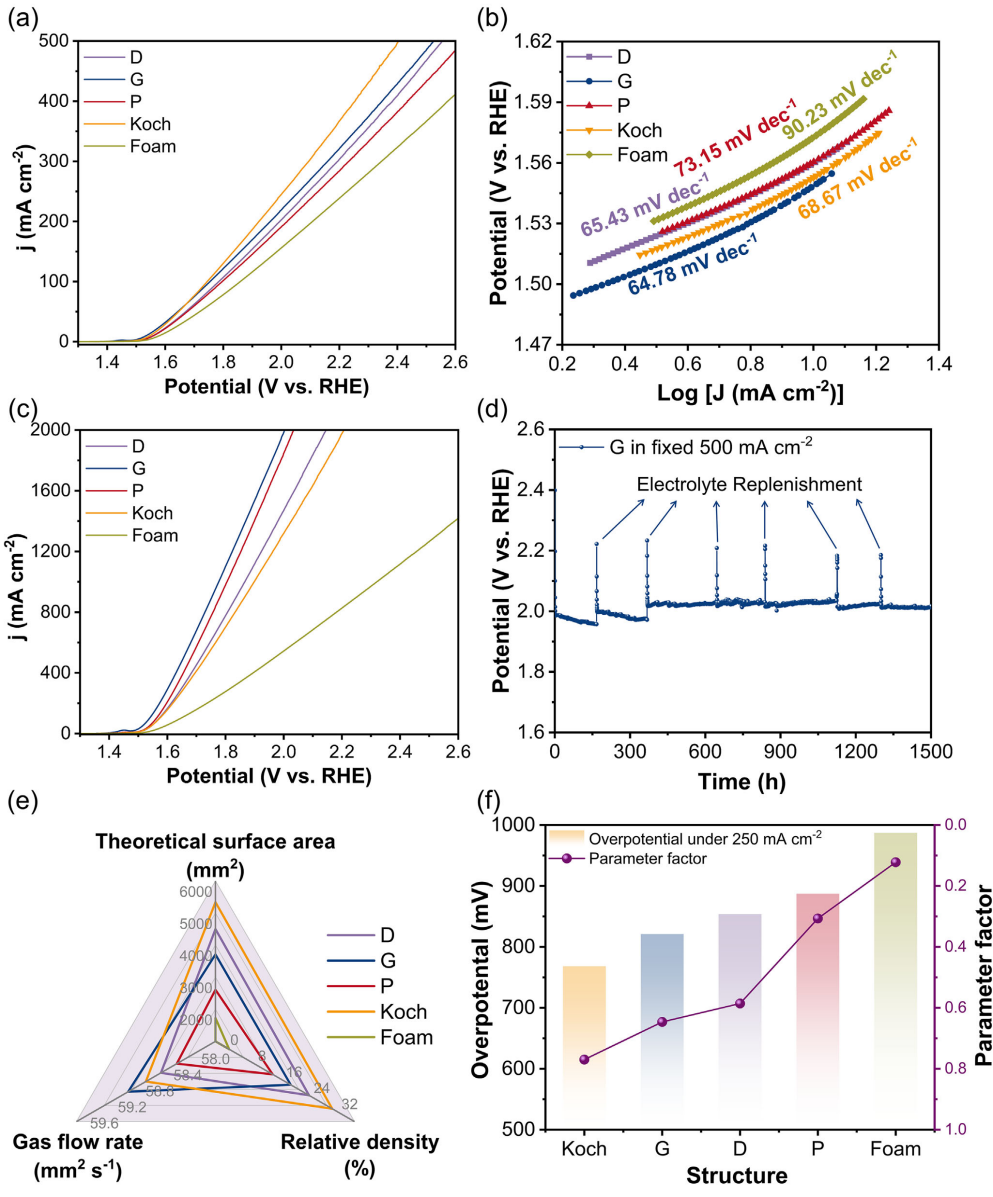
(a) LSV curves of different structures under projected area. (b) Tafel slopes of different structures. (c) LSV curves of different structures under cross‐sectional area. (d) Long‐term stability test of G structure. (e) Radar chart of three key factors (theoretical surface area, relative density, gas flow rate) for different structures. (f) Comparison chart of parameters with high surface‐area factor contribution and overpotential at 250 mA cm^−2^ in the three‐electrode system.

A radar chart was constructed using three key factors: the theoretical surface area, the relative density, and the gas flow rate (shown in Figure S11) measured at a current density of 250 mA cm^−2^, as illustrated in Figure 2e. This chart facilitates an intuitive comparison of the relationships between theoretical surface area and relative density, dominated by real surface area, vs. gas yield, dominated by mass transfer efficiency for the five structures. The ranking of the two surface‐area‐related factors from highest to lowest is as follows: Koch structure, D structure, G structure, P structure, and Foam structure. Higher surface areas typically expose more active sites. The yield of oxygen gas more accurately indicates the efficiency of bubble detachment along the electrode wall, signifying the mass transfer efficacy of the electrode structure. The G structure incorporates a continuous double‐helix flow channel, facilitating the expedited escape of bubbles generated at the surface along the wall [34]. Consequently, the G structure achieves a superior gas yield. A normalized scoring method was employed, utilizing the three properties from Figure 2e as proportional factors, and the outcomes were juxtaposed with the overpotentials at 250 mA cm^−2^ for various structures (Figure 2f). When the fluid property factor was below 33.33%, and the surface area factor exceeded 66.67%, the trend of the factor coefficients for all five structures was inversely proportional to the overpotential, meaning structures with higher parameters exhibited lower overpotentials. The results indicate that the magnitude of overpotential at high current densities is a synergistic outcome of surface area and mass transfer efficiency. In the three‐electrode system, the impact of geometric surface area clearly surpasses that of mass‐transfer efficiency, consistent with prior reports [35, 36]. Increased roughness and porosity raise the density of accessible active sites, lowering kinetic overpotential at a fixed current. However, when currents are normalized to the ECSA, the apparent advantage contracts markedly, indicating that most of the observed improvement originates from ECSA rather than from enhanced transport. Residual differences after ECSA normalization can be attributed to secondary effects, including local bubble coverage, double‐layer/ohmic contributions, and morphology‐dependent contact resistance, which modestly influence polarization at high rates. Our measurements reproduce these trends and further show that optimizing ECSA yields larger gains than altering flow conditions under identical electrolyte and stirring settings [37].

### 2.3 Electrochemical Performance of GDL in the AEMWE System

Figure 3a depicts the arrangement of the custom‐designed AEMWE, which includes end plates for solution input and output (A), collector plates for current input (B), runner plates for fluid input and output (C), the 3D‐printed TPMS structure serving as the anode gas diffusion layer and catalyst (D), platinum‐infused carbon paper functioning as the cathode diffusion layer and catalyst (E), and an intermediate anion exchange membrane (F). This design aligns more closely with the small modular configuration of industrial electrolytic cells and is appropriate for integrated systems with several electrolytic chambers [38]. The LSV curves calculated based on projected area are shown in Figure 3b, comparing the overpotential vs. current density relationship for each structure. Notably, the Koch structure, characterized by the maximum specific surface area, does not exhibit the most pronounced current density variation. At low current densities, its overpotential was higher than that of the G and D structures. After 3.1 V, due to the blockage caused by more bubbles, its overpotential was even higher than that of the P structure with the smallest surface area. Conversely, the G structure demonstrates the lowest overpotential across all current densities. This indicates that within the device, mass transfer effects outweigh surface area considerations, as bubble blockage reduces the number of active sites [39]. Consequently, enhancing bubble overflow is more crucial. The electrochemical impedance spectroscopy (EIS) diagram (Figure 3c) reveals a side‐by‐side structure of two semicircles, whose equivalent circuit consists of two resistor–capacitor pairs connected in series. The high‐frequency semicircle represents a relatively low charge transfer resistance (R_ct_), indicating enhanced catalytic activity of the electrode and accelerated reaction kinetics. The low‐frequency semicircle represents the membrane resistance (R_mt_), attributed to the resistance encountered by anions (OH^−^) migrating through the membrane at low frequencies. Combined with Figure 3d, the EIS diagram is decomposed into a comparison of the resistance distribution for each equivalent circuit element. Among these, the G structure exhibits the lowest charge transfer resistance, with a fitted value of (177.57 mΩ), more than half lower than that of the Foam structure's resistance with 393.78 mΩ, demonstrating its superiority in electron migration. Subsequently, the charge transfer resistance for the D structure is 210.40 mΩ, the P structure exhibited 221.86 mΩ, while the Koch structure demonstrated a charge transfer resistance of merely 326.58 mΩ, confirming that the obstructed bubbles hindered surface charge transfer. The membrane resistance signifies both the inherent resistance of the ion exchange membrane and the interface condition between the gas diffusion layer and the membrane. Utilizing the same membrane material, the 3D‐printed shell structure, characterized by its smooth boundary surfaces, offers an extensive contact area with the membrane. This facilitates the rapid adhesion of ions passed through the membrane to the electrode surface, thereby initiating reactions. Conversely, the Foam's mesh structure creates point‐contact areas with the membrane, which complicates ion migration and substantially elevates membrane resistance. Figure 3e presents LSV curves calculated using current density based on cross‐sectional area. The G structure demonstrates outstanding performance, exhibiting an overpotential of 310.28 mV at current density of 250 mA cm^−2^. As a pure nickel material, it already surpasses the catalytic efficiency of certain catalysts. Figure S3h presents the normalized LSV plot of the electrochemically active surface area (ECSA), revealing a consistent trend in the AEMWE system comparable to the three‐electrode system. This indicates that the electrochemical results are unaffected by surface area factors. The G structure demonstrates the lowest overpotential due to its greater active site mass at a low ECSA, while the P structure, possessing the lowest ECSA, exhibits superior activity performance compared to the D structure. Conversely, the Koch structure, characterized by the highest ECSA, experiences significant blockage from bubbles post‐reaction, resulting in the poorest electrochemical performance, even inferior to that of the Foam structure. The results for the 0.5 mm unit cell structures are shown in Figure S12. Among the three structurally similar configurations, the 2Koch structure with an average pore diameter below 50 µm exhibited the poorest performance, further confirming the disadvantage of ultra‐small pores.

**FIGURE 3:**
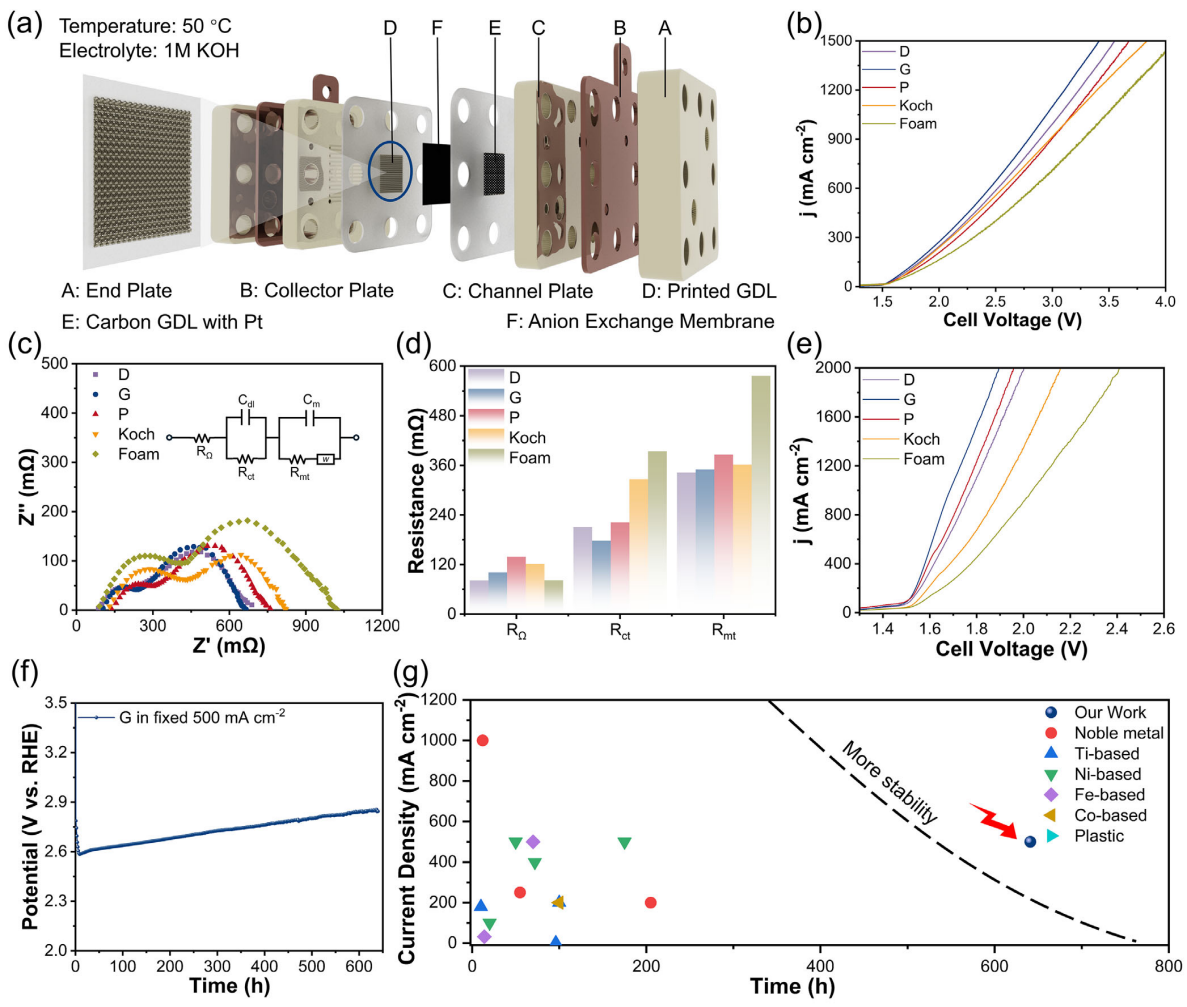
(a) The exploded view of an AEMWE system with 3D‐printed GDLs. (b) LSV curves of different structures under projected area. (c) EIS diagram of different structures in AEMWE system. (d) Resistance distribution across different structures of each equivalent circuit component. (e) LSV curves of different structures under cross‐sectional area. (f) Long‐term stability test of G structure and (g) Comparison with other works regarding stability over time and maintenance of current density at 50°C in 1 M KOH solution.

For the stability testing (Figure 3f), the G‐structured GDL still achieves 640 h of reaction stability. During the 640‐h test, the minimum cell voltage was 2.584 V, and the maximum cell voltage was 2.855 V, the voltage increased by 0.271 V, and the voltage decay rate had reached 10.5%, demonstrating less stable voltage changes compared to the three‐electrode system. The relatively low stability of AEMWE is attributed to the higher electrode surface corrosion capability of the flowing solution. The SEM image after failure (Figure S7b) reveals the impact of corrosion on the structure, with the boundaries and surfaces of the 3D‐printed GDL severely corroded but still retaining their basic morphological features. The reason for the shortened operating time compared to the three‐electrode system is explained in Supplementary Information. When compared with those in other studies (Figure 3g), the electrolysis cell system demonstrates significant stability advantages despite its reduced operating time [7, 9, 10, 11, 12, 13, 14, 17, 40, 41, 42, 43, 44, 45, 46]. Whilst many materials operate at high current densities, they exhibit extremely short operating times. Even those materials at low current densities fail to match our operating duration, highlighting the great potential of 3D‐printed GDLs in industrial applications. Figure S10 shows the LSV curves before and after stability testing, indicating a significant decrease in performance of the electrolytic cell after the reaction, and a certain performance decline was also observed after the GDL was tested with three electrodes alone, demonstrating the impact of surface corrosion.

### 2.4 Swift Permeation and Elevated Hydrophilicity of 3D‐printed GDLs

Contact angle measurement offers a good indication of a surface's hydrophilicity; more hydrophilic structures facilitate liquid movement towards the near‐surface region for reaction, even when internally filled with bubbles. Figure 4a depicts snapshots of a liquid droplet upon entry with pre‐wetted sample surfaces and after 0.1 s and 0.2 s of residence. Figure 4b presents the specific contact angle values for each structure, all of which demonstrate hydrophilic properties. The D structure initially displays a contact angle exceeding 25° upon droplet entry, which diminishes to below 15° after 0.2 s due to absorption. The G structure exhibits super‐hydrophilic properties with a contact angle below 10° and achieves complete wetting within 0.2 s. The P structure, due to the protruding hole geometry on its upper surface, presents a larger contact angle because of surface tension upon droplet application. However, after 0.2 s, the contact angle reduces to below 30° as the droplet permeates through the large pores. Conversely, the Koch structure, characterized by its reduced pore size and convoluted configuration, exhibits diminished hydrophilicity, remaining uninfiltrated even after 0.2 s, thereby demonstrating the lowest mass transfer rate. The Foam structure, with its irregular internal pore arrangement and polygonal mesh, elevates surface tension and obstructs subsequent droplet flow. Thus, despite being pre‐wetted, it presents a contact angle surpassing 40° and shows no flow even after 0.2 s. The results for the 0.5 mm unit cell structures (Figure S13) all exhibit slower wetting than the 1 mm unit cell structures, with structures failing to achieve complete wetting at 0.2 s, indicating unsuitable mass transfer efficiency for small apertures. Table 1 presents the roughness of printed surfaces with different structures, uniform surface chemical properties, and approximate roughness values that ascribe the differences in contact angles to mesoscale geometry rather than microscopic surfaces. Figure S14 illustrates the measurement results of the contact angle of underwater bubbles, which is approximately complementary to the contact angle of liquid in air, indicating the capacity of different structures to repel bubbles underwater and facilitate their separation more rapidly. Consequently, the low contact angle and minimal curvature of the G structure enhance the dynamic separation of bubbles.

**FIGURE 4:**
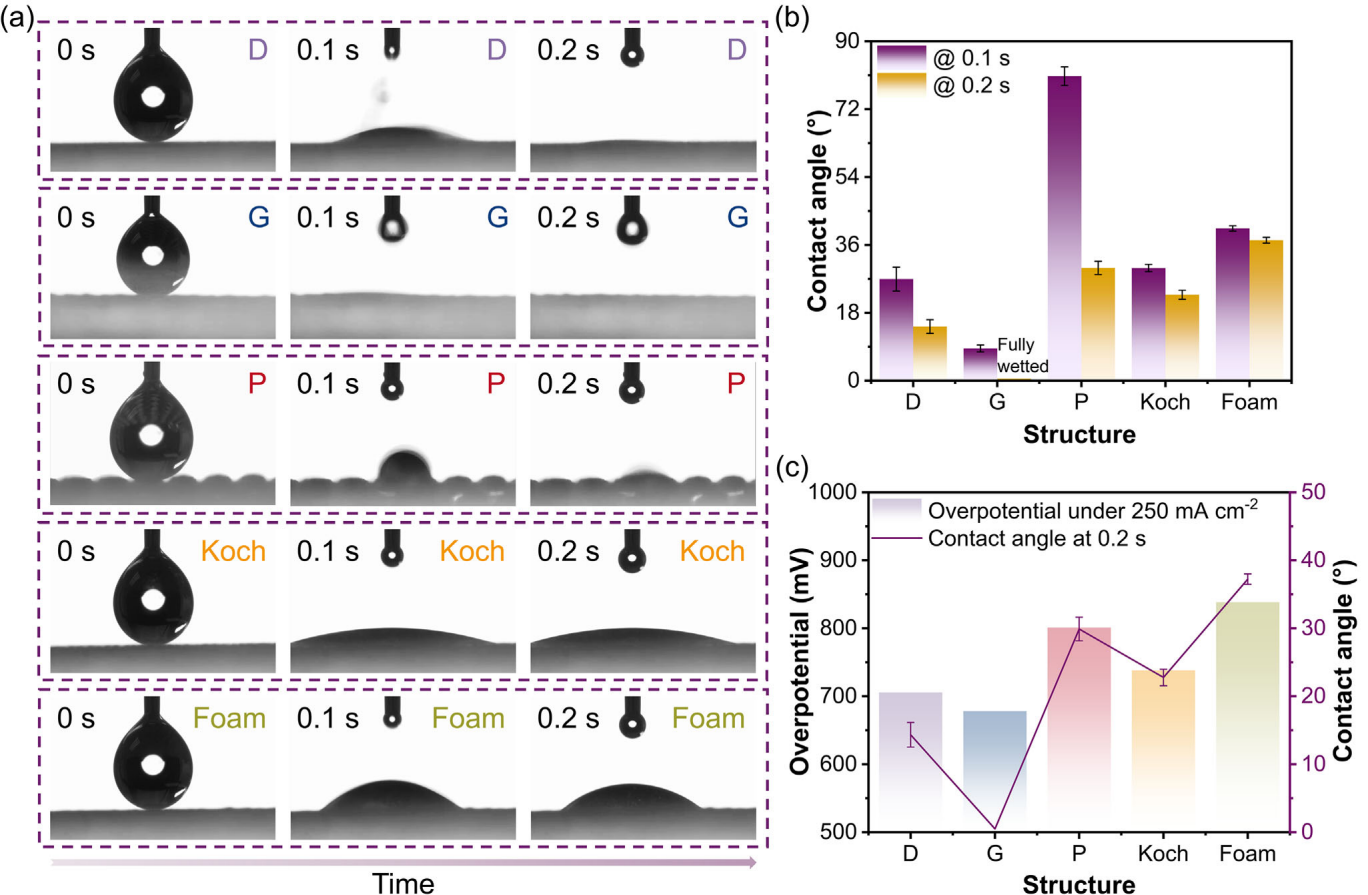
(a) Snapshots of contact angle for different structures at 0, 0.1, and 0.2 s droplet contact times. (b) Contact angle values for different structures. (c) Comparison of contact angles at 0.2 s and corresponding bubble flow rates for different structures.

**TABLE 1:** Sample names, basic structures, overall size, unit cell dimensions and finished wall thicknesses of different design structures.

Sample Name	Structure Function [A=2πa/L,A=X,Y,Z]	Relative Density [%]	Wall Thickness [µm]	Ave. Cross‐sectional Length [mm]	Surface Roughness Ra [µm]
D (Diamond)	$\phi_{D}\;\left(x,y,z\right)=\sin\left(X\right)\;\sin\left(Y\right)\sin\left(Z\right)+\sin\left(X\right)\cos\left(Y\right)\cos\left(Z\right)+\cos\left(X\right)\sin\left(Y\right)\cos\left(Z\right)+\cos\left(X\right)\cos\left(Y\right)\sin\left(Z\right)$	26.92	45.96	1199.44	1.35 ± 0.04
G (Gyroid)	$\phi_{G}\;\left(x,y,z\right)=\cos\left(X\right)\;\sin\left(Y\right)+\cos\left(Y\right)\sin\left(Z\right)+\cos\left(Z\right)\sin\left(X\right)$	21.63	45.68	972.62	1.60 ± 0.025
P (Primitive)	$\phi_{P}\;\left(x,y,z\right)=\cos\left(X\right)+\cos\left(Y\right)+\cos\left(Z\right)$	16.46	49.44	754.84	1.80 ± 0.053
Koch (Fischer‐Koch S)	$\phi_{\mathrm{Koch}}\;\left(x,y,z\right)=\cos\left(2X\right)\;\sin\left(Y\right)\cos\left(Z\right)+\cos\left(X\right)\cos\left(2Y\right)\sin\left(Z\right)+\sin\left(X\right)\cos\left(Y\right)\cos\left(2Z\right)$	33.60	43.94	1690.48	1.75 ± 0.058

Figure 4c depicts the correlation between the contact angle at 0.2 s and the overpotential at 250 mA cm^−2^, highlighting the effect of mass transfer efficiency on the electrochemical performance of the electrolytic cell. The G structure demonstrates a super‐hydrophilic surface that achieves complete wetting at 0.2 s, exhibiting a reduced overpotential of 678.08 mV at 250 mA cm^−2^, attributable to the minimal contact angle between the surface and water during the reaction. Bubbles form with an extensive contact area with the surface, resulting in a spherical crown shape with weak adhesion. Consequently, bubbles detach more easily under fluid disturbances and buoyancy forces, facilitating the removal of bubbles and exposing additional active sites for reaction participation, thereby diminishing concentration polarization overpotential and allowing electrolysis at lower voltages. At a current density of 250 mA cm^−2^, structures with lower contact angles generally show lower overpotentials. This suggests that in the electrolytic cell system, the increased exposure of active sites from accelerated bubble desorption carries more weight than the electrode's intrinsic surface area. As a result, mass transfer efficiency is more important in determining electrochemical performance. Simultaneously, all structures demonstrate considerable hydrophilicity, with liquid absorption occurring within one second. Consequently, the variation in hydrophilicity is a secondary factor affecting Ohmic resistance; thus, a consistent correlation is absent, with the influence of contact and structural characteristics prevailing.

### 2.5 Mass‐transfer‐Dominated Fluid Characteristics in 3D‐printed GDLs

To further demonstrate that mass transfer efficiency is the principal determinant of the electrochemical performance of GDLs in the electrolysis cell, the volumetric flow rate of oxygen evolution was quantified using the drainage method for four printed structures. The comparison of volumetric flow rate against overpotential at a current density of 250 mA cm^−2^ is depicted in Figure 5a. Overall, the oxygen flow rate demonstrates an inverse correlation with overpotential, indicating that structures with elevated flow rates exhibit reduced overpotentials. The G structure displays the highest volumetric flow rate due to its mass transfer efficiency via the double‐helix surface, which allows for effective bubble expulsion after generating a substantial volume, while concurrently facilitating solution access to the surface reaction and mitigating concentration polarization, thus achieving the lowest overpotential. In contrast, the P structure enabled bubble escape post‐desorption due to its large pores, but its limited surface area resulted in a high overpotential. The Koch structure exhibited the lowest flow rate due to significant bubble clogging, with the ensuing concentration polarization negating its overpotential advantage within the electrolytic cell.

**FIGURE 5:**
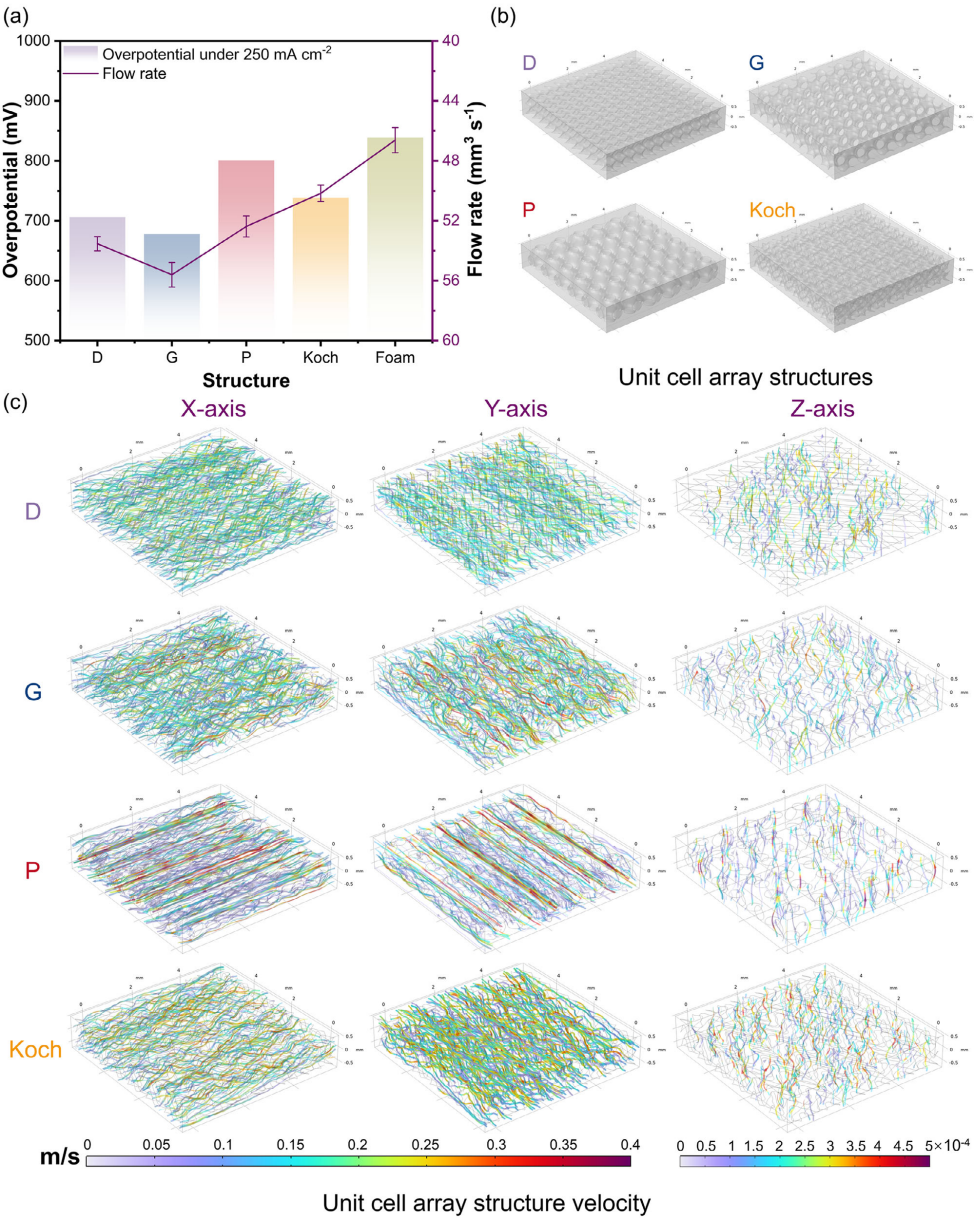
(a) Comparison of volumetric flow rate vs. overpotential at a current density of 250 mA cm^−2^. (b) Schematic diagram of unit cell array structure modelling. (c) Velocity variation diagram of the unit cell array structure along the x, y, and z axes.

To analyze the internal two‐phase flow dynamics and model the pressure differential between the outlet and the gas generation interface, the two‐phase flow simulation of the internal unit cell array structure was integrated with the free and porous medium flow simulation of the overall homogenized structure. Figure 5b illustrates the unit cell array modelling for four distinct TPMS structures, in addition to a homogeneous structure devoid of lattice configuration. Each structure consists of a 6×6×1 unit cells model to enable permeability homogenization along the x and y axes, while a single‐layer TPMS structure functioning as the inner wall is supplied with a gas‐liquid mixture from the boundaries along the x, y, and z axes. Figure 5c depicts velocity streamlines within the unit cell arrays of the four TPMS structures. The D structure demonstrates a consistently low flow velocity due to the excessive number of sharp corners and edges that cause fluid blockages, in conjunction with the small internal orifices that impede the establishment of stable flow regions. The flow in the G structure is more uniform, exhibiting minimal velocity variation; the helical streamlines along the wall face no obstructions, preserving consistent velocity and effectively transporting bubbles from the wall surface. In contrast, the P structure demonstrates elevated flow velocities concentrated in the central interconnected open‐pore region, while the streamlines adjacent to the wall exhibit diminished velocities due to decreased flowability at the wall surface, thus hindering bubble entrainment. The flow patterns in the Koch structure are more chaotic, with its intricate walls resulting in uneven velocity distribution throughout the interior; additionally, the reduced velocity at the outlet significantly hampers mass transfer efficiency.

Figure 6a displays a homogenized model combining the laminar flow region of the serpentine channel with Darcy flow in the porous region of homogeneous permeability. Multiphysics simulation was performed to determine the pressure drop between the outlet and the bubble inlet. Figure 6b shows the homogenized velocity streamlines within the electrolysis cell after the permeability of the four structures was homogenized. Structures G and P, characterized by higher permeability and porosity, cause the liquid to flow more readily into the GDL layer. Simultaneously, the mixed fluid within the GDL layer tends to circulate internally within the structure, creating favorable conditions for bubble entrainment. In contrast, the Koch structure directs fluid flow primarily through the channels without entering the GDL layer. This reduces both the inflow of reaction solutions and bubble entrainment, resulting in poorer electrochemical performance. Figure 6c illustrates the internal pressure variations across the four configurations. The predominant trend shows elevated pressure at the liquid inlet and reduced pressure at the gas‐liquid mixture outlet. Configurations G and P exhibit lower inlet pressures, owing to their reduced flow resistance facilitating easier liquid ingress into the GDL layer. Conversely, the Koch configuration demonstrates markedly higher‐pressure values, as its lower permeation rate impedes liquid penetration into the GDL layer. Figure 6d illustrates the relationship between simulated pressure drop and measured bubble outlet flow rate across the four structures. Pressure drop and flow rate exhibit an approximate correlation, with structures featuring lower pressure drops demonstrating higher outlet bubble yields. The distinction lies in the lower flow rate of the P structure at its reduced pressure drop compared to that of the D structure. This occurs because the P structure possesses a lower surface gas production rate; consequently, despite its higher mass transfer efficiency, its flow rate cannot match that of the higher‐yielding D and G structures. The Koch structure's catastrophic mass transfer performance means that, despite higher gas production in the electrolysis cell, its gas flow rate is reduced in the electrolytic cell due to its disastrous mass transfer capability. Combining simulation results with gas production data demonstrates that, in industrial electrolytic cells, the mass transfer capability of GDLs exerts a greater influence on electrochemical performance than apparent surface area. The pressure drop indicated by the simulation result is challenging to quantitatively validate through experiments. Figure S15 correlates the simulated outlet flow velocity with the experimentally measured outlet flow velocity, demonstrating a significant correlation between the two results and endorsing the use of current Multiphysics models to assess pressure drop variations in gas diffusion layers. The relationship between Faraday efficiency and gas flow rate is presented in Figure S16. Notably, the G‐structured electrode achieves a Faraday efficiency of 97.3%, indicating that nearly all of the applied charge is effectively utilized for oxygen generation.

**FIGURE 6:**
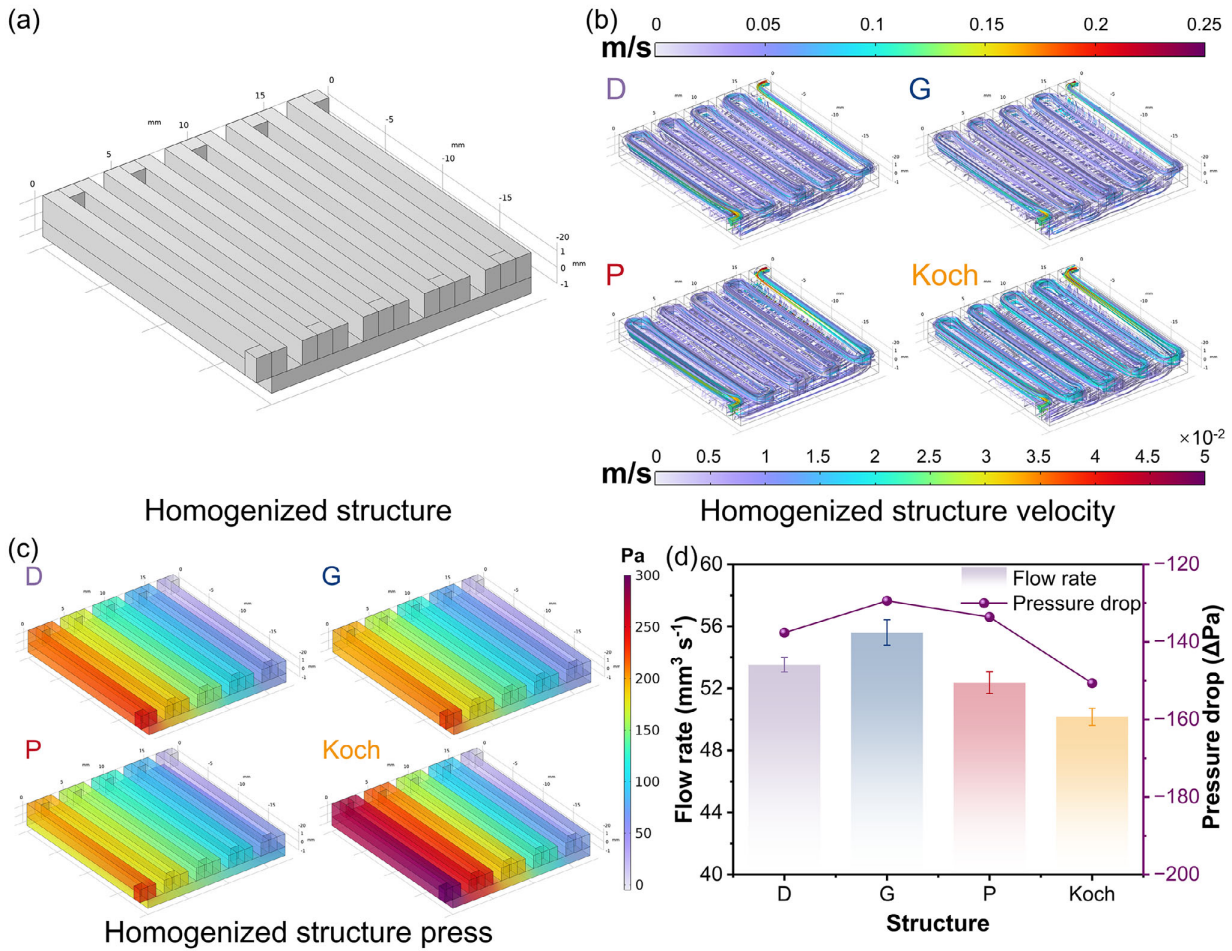
(a) Schematic diagram of a homogenized structure electrolytic cell. (b) Velocity distribution diagram of the homogenized structure (The legend above indicates flow channel velocity; the legend below indicates flow velocity within the porous structure). (c) Pressure variation diagram for a homogeneous structure. (d) Comparison of average pressure drop and flow rate across different structures.

### 2.6 Further Discussion on the Key Influencing Factors

The normalized weighted scoring method simplifies qualitative assessment of the significance of real surface area and mass transfer capacity in GDL design, offering insights for tailored GDL configurations. Among the key factors of the AEMWE system depicted in Figure 7a, theoretical surface area and relative density pertain to real surface area, with the four structures ranked as Koch, D, G, and P in descending order. The contact angle, indicative of a material surface's affinity and hydrophobicity towards gas and liquid, is essential to the mass transfer factor. The gas flow rate denotes both the volume of gas produced and the ability of bubbles to detach from the gas diffusion layer, illustrating mass transfer efficiency. Pressure drop is commonly employed in simulations to measure gas obstruction within the fluid system during gas‐liquid two‐phase flow, serving as a vital metric for evaluating bubble discharge dynamics. The G structure consistently demonstrates the most robust mass transfer capability, whereas the Koch structure is comparatively the weakest. When the weight of factors pertaining to real surface area is below 43.46%, and the weight of factors concerning mass transfer capacity exceeds 56.54%, the parameter factors of the four structures demonstrate a negative correlation with overpotential, indicating that structures with higher scores exhibit lower overpotential. Conversely, when the weight of factors related to real surface area drops below 24.46%, the factor weight of the P structure surpasses that of the Koch structure. Figure 7b shows the relationship diagram when the real surface area weight is 40%, where the ranking exhibits a negative correlation with overpotential. Under this weight, the contribution ratios vary among structures: The G structure, possessing the highest mass transfer capability, contributes 19.02% to the total score based on its real surface area. In contrast, the real surface area of the D structure accounts for 42.65% of its score, indicating a greater contribution compared to that of the G structure. The Koch structure, despite its substantial real surface area contribution of 81.97%, attains a score of only 0.488, which is lower than that of structures with superior mass transfer contributions. Although the P structure has no contribution due to its minimal real surface area, its mass transfer contribution ranked third, yielding a composite score of 0.242. Consequently, the G structure, with its superior mass transfer efficiency, represents the optimal TPMS configuration for electrolytic cell applications. This scoring method provides a concise summary of the key attributes in 3D‐printed GDL structures, offering valuable guidelines for the future TPMS lattice GDL designs. Although comparisons between the “mass‐transfer factor” and the “surface‐area factor” are scarce, both exert first‐order control over electrolysis cell performance, and several studies imply that transport often dominates under practical loads. Specifically, designs that intensify bubble removal, shorten diffusion paths, and lower two‐phase resistance can outperform larger‐area structures when current density is high and gas holdup becomes rate limiting. For example, architectures that constrain bubble size and promote uniform detachment achieve higher currents at the same voltage despite using only a fraction of the geometric area, evidencing a net gain from improved transport rather than added surface [47]. Likewise, reaction‐engineering strategies that enhance reactant/product conveyance reduce overpotential even when the nominal area is curtailed, highlighting the primacy of mass‐transfer control [48]. Additively manufactured GDLs with increased porosity and optimized pore connectivity further demonstrate lower charge‐transfer resistance and higher current density due to superior gas diffusion and water management [49].

**FIGURE 7:**
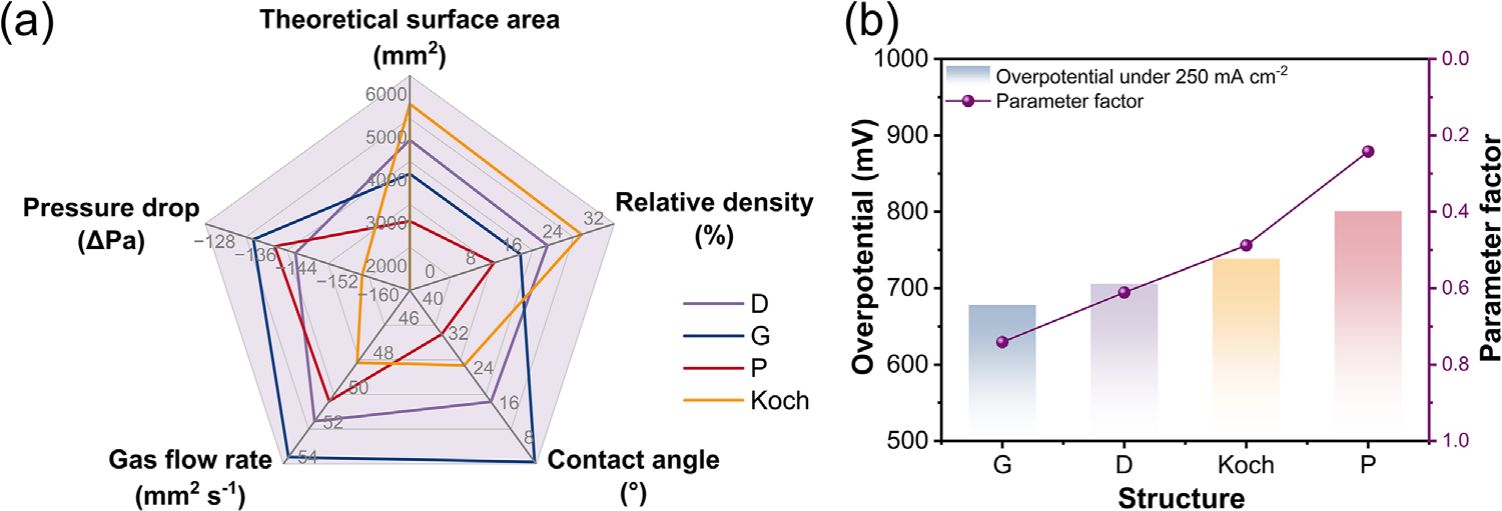
(a) Radar chart of five key influencing factors (theoretical surface area, relative density, contact angle, gas flow rate, pressure drop) for different structures. (b) Comparison chart of parameters with 60% fluid factor contribution and overpotential at 250 mA cm^−2^ in the AEMWE system.

## 3 Conclusions

In summary, thin‐walled TPMS lattice nickel GDLs were fabricated using micro‐LPBF combined with STL‐free laser toolpath printing. Four TPMS structures (D, G, P, and Koch) were designed and evaluated for electrochemical performance in both three‐electrode and industrial electrolytic cell systems. 3D‐printed Gyroid lattice structures show the highest gas flow rate with lower Tafel slope (64.78 mV dec^−1^) than foam nickel and maintain operation for 1500 h at a high current density of 500 mA cm^−2^. The Koch structure demonstrated a lower overpotential due to higher proportion of real surface area. In contrast, the G structure exhibited superior performance in the electrolytic cell system and a resistance value of 177.57 mΩ, maintaining stability for 640 h at 500 mA cm^−2^. The results from the three‐electrode system were attributed to surface area‐dominated high reaction zones, while those from the industrial cell system were linked to mass transfer efficiency‐dominated bubble detachment. To validate this phenomenon, contact angle measurement and CFD simulations were performed, which confirmed that the G structure, demonstrating the most exceptional mass transfer capability, had the lowest contact angle. Simulation results illustrated its smoothest velocity streamlines and lowest pressure drop, establishing the foundation for its electrochemical performance in the AEMWE system. Multi‐factor normalized weighted scoring analysis indicates that mass transfer capability exerts a greater influence on electrochemical performance than real surface area within the AEMWE system. These GDLs exhibited significantly enhanced stability and OER performance compared to commercial foam nickel structures in industrial AEMWE cells, which renders them the ideal selection for nascent rule‐based GDLs.

## 4 Methods

### 4.1 Design of GDLs and Production With Micro‐LPBF

The TPMS structures in the GDLs include Primitive (P), Gyroid (G), Diamond (D), Fischer–Koch S (Koch), with length and width dimensions of 20 mm, which were designed as a single cell with a thickness of 1 mm in text, a double 0.5 mm thickness cell in Supplementary Information to make a scale comparison, which are called 2P, 2G, 2D and 2Koch. The implicit modeling of TPMS structures was created by surface functions in MATLAB software (Version R2022a, MathWorks Inc) [50]. The specific parameters of the designed structures are shown in Table 1. All samples were printed by the self‐developed micro‐LPBF Han's Laser M100µ, using a Yb laser source (λ = 1.07 µm) with a beam size of 25 µm. Nickel powder of 99.9% purity was produced using gas atomization to form spherical particles with a diameter of 5–25 µm. N_2_ gas was used to maintain an inert atmosphere during the printing process, with an O_2_ content of less than 500 ppm. STL‐free single paths for each layer were generated by direct slicing, with each segment having a path length of 100 µm and a spacing of 30 µm to form paths of suitable intensity. The energy density is calculated by Equation (1):

(1) $$E=\frac{P}{v\;\times h\times t}$$

where *E* is energy density, *P* is the laser power, *v* is the scanning speed, *h* is the hatch distance, *t* is the layer thickness. The energy window in which *E* is located is the key to optimizing the printing parameters, and the nickel‐based printing parameters obtained after several experiments were optimized and applied to the printing of these samples [51]. The laser power is set to 60 W, with a scanning speed of 1000 mm s^−1^, hatch distance of 0.05 mm, and layer thickness of 0.01 mm, tailored for single‐path micro‐printing. The calculated energy density is 120 J mm^−3^. The wall thickness and surface roughness were assessed utilizing a Hirox RH‐2000 digital 3D microscope. Average values were derived from three regions of each of the five samples. Wall thickness was determined as the thickness of a single path on the uppermost surface, whereas surface roughness was evaluated in the powder‐free area of the saddle surface, indicative of the surface process of the printed material.

### 4.2 Electrochemical Measurements in a Three‐Electrode System

The printed GDLs were electrochemically tested in a sealed three‐electrode system, which was ultrasonicated for 10 min and immersed in 100 mL 1 M KOH for 10 min to remove the surface oxide layer before measurement. The 3D‐printed Ni electrode and the nickel foam (Foam) with the same thickness were used as working electrodes, with the electrode sheet facing the counter electrode in order to approximate the electrode reaction area as a planar area. Figure S17 shows the performance comparison between Foam and other engineered PTLs, projected‐area normalized LSV curve show Ni felt and Ni foam exhibit similar current densities. Despite higher ECSA, Ni felt does not outperform foam because the latter's larger holes enhance mass transfer and expose more active sites. ECSA‐normalized LSVs further reveal lower currents for Ni felt than Ti felt, implying greater transport resistance and underutilized sites. Overall, commercial Ni foam remains the most effective PTL. Carbon rods and mercury‐mercury oxide electrodes (Hg/HgO) were used as counter electrodes and reference electrodes, respectively, and 1 M KOH was used as the electrolyte, and the LSV tests with a scan rate of 5 mV s^−1^, the Tafel slope tests with a scan rate of 1 mV s^−1^, and EIS tests under 100 mA cm^−2^ and stability tests under 500 mA cm^−2^ were performed by the Corrtest CS310 electrochemical workstation at room temperature. ECSA was measured by double‐layer capacitors, CV tests were conducted at 10, 20, 30, 40, 50 mV s^−1^ scan speeds under voltages ranging from 0 to 0.1 V. Stability testing of each system was performed utilizing the constant current technique at a current density of 500 mA cm^−2^, with a recording interval of 1 min.

### 4.3 Assembly of AEMWE Cell and Electrochemical Measurements

A self‐designed AEMWE cell for measuring GDL performance is shown in Figure 3. The overall structure consists of symmetrical titanium end plates (thickness 15 mm), runner plates (thickness 10 mm) and copper collector plates (thickness 2 mm) with serpentine‐shaped runners to match the liquid input and gas discharge. A serpentine flow channel is provided to match the liquid inlet with the gas outlet. Equal‐thickness PTFE gaskets were placed between the two runner plates to secure the GDL. Printed nickel‐based TPMS structures were used as the anode GDL and compared with 1 mm thick Foam, and a carbon paper with a Pt loading of 0.1 mg cm^−2^ was employed as the cathode GDL. Fumasep FAB‐PK‐130 membrane was used as the anion‐exchange membrane. The assembly pressure of the fixtures was set to 5 N m. The projected area of the electrodes was 4 cm^2^. The temperature of the electrolytic cell was maintained at 50°C using a heating rod, whilst ensuring the beaker remained insulated. 500 mL 1 M KOH was used as the electrolyte, with the electrolyte delivered by a peristaltic pump at a rate of 200 mL min^−1^. Electrolyte replenishment was conducted every 48 h due to the consumption under high temperature.

### 4.4 Electrochemical Analysis

In this case, the correction relation for the conversion of the test potential to the reversible hydrogen electrode (RHE) potential using Hg/HgO as the reference electrode is given by Equations (2) and (3):

(2) $$E_{\mathrm{RHE}}=E_{\mathrm{Hg}/\mathrm{HgO}}+\frac{2.303\mathrm{RT}}{F}\cdot \mathrm{pH}+E_{\mathrm{Hg}/\mathrm{HgO}}^{\Theta }$$

At a temperature of 25°C,

(3) $$E_{\mathrm{RHE}}=E_{\mathrm{Hg}/\mathrm{HgO}}+0.059\cdot \mathrm{pH}+0.098\;V$$

where the *E_RHE_* is the potential when using RHE as the reference electrode, the *E_Hg/HgO_* is the potential under Hg/HgO as the reference electrode, *E^θ^_Hg/HgO_* is the potential difference between Hg/HgO as a reference electrode at 25°C and the standard normal hydrogen electrode (NHE), which is usually considered to be 0.098 V.

The overpotential is the difference between the actual potential of the electrochemical reaction and the equilibrium potential, which is given by Equation (4):

(4) $$\eta =E_{i}\;-E_{t}$$

where *η* is the overpotential, *E_i_* represents the potential of the electrode, *E_t_* is the potential at equilibrium.

The Tafel slope was calculated using Equation (5):

(5) $$\eta =-\left(\frac{2.303\mathrm{RT}}{\alpha F}\right)\log\left(i_{0}\right)+\left(\frac{2.303\mathrm{RT}}{\alpha F}\right)\log\left(i\right)=a+\mathrm{blog}\left(i\right)$$

where *i_0_* is the exchange current density, *α* is the charge transfer coefficient, *F* is the Faraday constant, *R* is the ideal gas constant and *T* is temperature.

The overall print area is 20 × 20 mm^2^, and this projected area is used as the corresponding calculation area for current density and resistance density, which facilitates data alignment between different studies. However, this is not fair to the TPMS with low wall thickness. The large aperture brings the advantage of bubble transport and also has higher current density when the average cross‐sectional area is used as the calculation area, which also accurately reflects the actual reactivity and realistically reflects the exposure of the active site. The average cross‐sectional length, derived from the summation of individual layers as illustrated in Table 1, is multiplied by the wall thickness of each structure to get the average cross‐sectional area, henceforth referred to as the cross‐sectional area in this study. For GDL with complex structures, it is necessary to carry out a comparison of both areas as current density areas [52].

### 4.5 Model Design and CFD Simulation

STL model files for different TPMS structures were created using MATLAB code. However, the mesh generated in this way was irregular, making it difficult to import into simulation software to generate surfaces. Therefore, the STL files were imported into HYPERMESH for remeshing to produce regular surface patches, which were arrayed for simulation. Using COMSOL software, a microstructure was modeled for GDL. Six unit cells were constructed along the x‐axis and y‐axis respectively to serve as sources for the homogenized permeability parameters, while one unit cell was constructed along the z‐axis to accommodate the single‐layer structure, and a 6.2 × 6.2 × 1.2 mm^3^ cube was constructed to serve as the bubble flow region for the unit cell array structure. Benefiting from the thin‐walled structure achieved through single‐path printing, the inner walls of the single‐layer structure could be incorporated during microstructure generation. This significantly reduced the number of mesh elements, thereby enabling the simulation of multi‐TPMS structures. Bubble flow simulations were conducted along the x, y, and z axes for the microstructure region. The average gas‐liquid ratio results from the bubble flow simulation (Figure S18) were imported into the unit cell array simulations. The flow velocities along the x and y axes were set to 11.1111 cm s^−1^, derived from measured flow rates. The z‐axis velocity was calculated as 0.014815 cm s^−1^, which is the average gas production rate of the four structures. A GDL homogenization layer measuring 17 × 20 × 1 mm was established as the maximum serpentine flow zone, and a 1 × 1.5 mm serpentine flow channel was constructed to build homogenized structure. A Multiphysics simulation was performed for both free and porous medium flows, with laminar flow in the channel region and Darcy's law‐dominated flow in the porous material zone. The inlet velocity at the serpentine channel entrance was determined by conversion to 11.1111 cm s^−1^. The porosity, anisotropic permeability, and Z‐axis inlet velocity of the porous matrix are detailed in Table 2. The permeability k is obtained from Equation (6):

(6) $$k=\frac{\mu \mathrm{QL}}{\Delta \mathrm{PA}}\;$$

where *µ* is the fluid viscosity, *Q* is the flow rate per unit time, *L* is the length of the structure, *ΔP* is the pressure difference across the structure, *A* is the cross‐sectional area.

**TABLE 2:** Parameters of different structures in Darcy's law‐dominated flow.

Sample Structure	Porosity	X‐axis Permeability [×10^−5^ cm^2^]	Y‐axis Permeability [×10^−5^ cm^2^]	Z‐axis Permeability [×10^−5^ cm^2^]	Inlet Velocity [cm s^−1^]
D	0.7308	3.09607	3.07710	2.65456	0.014695
G	0.7837	3.46180	3.86600	3.77900	0.014815
P	0.8354	5.16066	5.15687	0.38949	0.014640
Koch	0.6640	1.85576	1.86620	1.40330	0.014750

## Author Contributions

**Tianxiao Niu**: Writing – original draft, visualization, methodology, investigation, data curation. **Rui Li**: methodology, software, writing – review and editing. **Tianyu Gao**: methodology, visualization. **Deyong Sun**: software, formal analysis. **Xu Song**: writing – review and editing, supervision, project administration, resources, funding acquisition. **Xiao Chen**: data curation, conceptualization, writing – review and editing, supervision. **Junhao Ding**: conceptualization, visualization, methodology, writing – review and editing. **Shuo Qu**: methodology, validation, resources.

## Conflicts of Interest

The authors declare no conflicts of interest

## Supporting information




**Supporting File**: advs77317‐sup‐0001‐SuppMat.docx.

## Data Availability

The data that support the findings of this study are available from the corresponding author, Dr. Xu Song, upon reasonable request.
