# Supplementary material for: Revealing the Underlying Mechanisms in Performance Enhancement of Shellular Lattice Electrodes During Anion Exchange Membrane Water Electrolysis Process
Source: Adv Sci (Weinh). 2026 Aug 21:e77317. Online ahead of print. doi: 10.1002/advs.77317 (PMC13496155; doi:10.1002/advs.77317)
Supplement: Supplementary file 1 [file ADVS-9999-e77317-s001.docx]

Supplementary Information

[Revealing the Underlying Mechanisms in](https://scholar.google.com/citations?view_op=view_citation&hl=en&user=3Akl_AcAAAAJ&sortby=pubdate&citation_for_view=3Akl_AcAAAAJ:9Nmd_mFXekcC) Performance Enhancement of Shellular Lattice Electrodes during Anion Exchange Membrane Water Electrolysis Process

Tianxiao Niu, Rui Li, Xiao Chen*, Deyong Sun, Tianyu Gao, Junhao Ding, Shuo Qu*, Xu Song*

1. **Determining the boundary conditions and gas-liquid ratio in unstructured bubble flow**

The boundary conditions are established as follows: in the bubble flow simulation (laminar flow, Re<2000), the inlet of the serpentine channel and the interface between the gas diffusion layer and the membrane are designated as inlets, while the opposite end of the serpentine channel is designated as an outlet. The remaining surfaces are defined as no-slip walls. To replicate real-world conditions, the liquid inlet velocity is set to a measured value of 11.11 cm s^-1^, and the bubble inlet is approximated by the measured average gas generation rate from **Figure** S11, which is 58.90 mm^3^ s^-1^. This value more closely approximates the gas production rate at the theoretical current density of 250 mA cm^-2^. Assuming the entire base surface serves as the gas production surface, resulting in an incoming gas velocity of 0.014725 cm s^-1^, with a gas flux of 1.46×10^-4^ kg m^-2^ s^-1^. Simulation results are shown in **Figure** S18, where blue streamlines represent liquid flow and green streamlines denote bubble flow. Liquid first spreads fan-shaped across the entire domain plane before flowing to the outlet, while bubbles move towards the outlet along the bottom plane. **Figure** S18(c) and (e) display the volume fraction of internal dispersed phase bubbles. As the primary bubble flow region resides within the GDL layer, the average gas volume fraction obtained beneath each GDL layer plane can be approximated by substituting the inlet bubble volume fraction into the crystal lattice arrangement structure. The bubble volume fraction in the XZ and YZ planes is 0.2046. For the XY plane, which is the primary gas flow plane, the substituted proportion is the bubble volume fraction of the inlet plane, which is 0.5187. The average volume fraction of internal dispersed phase bubbles and the average flow velocity in three directions were incorporated into the unit cell array model. Six distinct TPMS unit cells were fabricated along the x-axis and y-axis to serve as sources of uniform permeability parameters, while a unit cell was created along the z-axis to accommodate a single-layer structure. Additionally, a cube measuring 6.2 × 6.2 × 1.2 mm^3^ was established as the bubble flow area of the unit cell array structure. The X, Y, and Z axes each feature one inlet and one outlet, with the remaining surfaces designated as wall surfaces, and various TPMS structures were internally integrated and designated as inner walls. Assuming minimal variation in internal flow velocity, the gas-liquid mixed flow velocity in the bubble flow simulation is designated as the inlet flow velocity for the X and Y axes, while the Z-axis velocity is computed as 0.014815 cm s^-1^. This value results from converting the average gas flux based on the gas production area. The anisotropic permeability across the three axes was determined using a specific formula, with porosity and permeability employed as parameters of the porous medium to simulate the flow dynamics in both free and porous media across multiple physical fields. The flow channel area is designated as a laminar flow region, while the gas diffusion layer area is characterized as a Darcy's law porous medium flow. Various TPMS structures are represented by altering the porosity, permeability, and gas inlet flow rate of the porous medium. The flow channel area is designated as a laminar flow region, while the gas diffusion layer is characterized as a porous medium flow according to Darcy's law. Various TPMS structures are differentiated by altering the porosity, permeability, and gas inlet flow rate of the porous medium. The Z-axis gas inlet velocity is derived from the gas production of the three-electrode system with distinct TPMS structures, as illustrated in **Figure** S11, which aligns with the surface gas production at a current density of 250 mA cm^-2^. The mass transfer capacity of the four structures is quantitatively assessed by comparing the pressure drop between the outlet and gas inlet.

2. **Principles and Formulas of Fluid Simulation**

The bubble flow model in COMSOL is a simplified Euler-Euler model, requiring the solution of only one continuity equation, one momentum equation, and the gas phase volume fraction transport. The momentum equation is given by:

$$\begin{aligned} \varphi_{l}\rho_{l}\frac{\partial u_{l}}{\partial t}+\varphi_{l}\rho_{l}\left( u_{l}\cdot\nabla\right)u_{l}=-\nabla p+\nabla\cdot\left[ \varphi_{l}\left( \mu_{l}+\mu_{T} \right)\left( \nabla u_{l}+\nabla{u_{l}}^{T}-\frac{2}{3}\left( \nabla\cdot u_{l} \right)I \right) \right]+\varphi_{l}\rho_{l}g\left( 1 \right) \end{aligned}$$

where *u_l_* is the velocity vector, *p* is the pressure, *φ* is the phase volume fraction, *ρ* is the density, *g* is the gravity vector, *μ_l_* is the dynamic viscosity of the liquid, *μ_T_* is the turbulent viscosity. The subscripts “l” and “g” denote quantities related to the liquid phase and the gas phase, respectively.

The continuity equation is:

$$\begin{aligned} \frac{\partial}{\partial t}\left( \varphi_{l}\rho_{l}+\varphi_{g}\rho_{g} \right)+\nabla\cdot\left( \varphi_{l}\rho_{l}u_{l}+\varphi_{g}\rho_{g}u_{g} \right)=0\#\left( 2 \right) \end{aligned}$$

and the gas phase transport equation is:

$$\begin{aligned} \frac{\partial\varphi_{g}\rho_{g}}{\partial t}+\nabla\cdot\left( \varphi_{g}\rho_{g}u_{g} \right)=-m_{gl}\#\left( 3 \right) \end{aligned}$$

where *m_gl_* is the mass transfer rate from gas to the liquid.

For the flow through free and porous media within a homogeneous structure. The porous medium employs the Darcy flow model, with the equation simulating flow within the porous domain being:

$$\begin{aligned} \frac{\partial}{\partial t}\left( \epsilon_{p}\rho\right)+\nabla\cdot\left( \rho u \right)=Q_{m}\#\left( 4 \right) \end{aligned}$$

$$\begin{aligned} u=-\frac{\kappa}{\mu}\nabla p\#\left( 5 \right) \end{aligned}$$

where *κ* is the permeability, *ε_p_* is the porosity.

3. **Normalized weighted scoring method**

Max-min normalization adjusts the numerical range of factor data from different structures to [0, 1], where the maximum value is 1 and the minimum is 0. Other data points are linearly scaled relative to this reference. The average of scores within the same category is then multiplied by the weight to obtain the composite score for a single structure. Specifically, for a given factor value:

$$\begin{aligned} X_{n}=\frac{x-x_{min}}{x_{max}-x_{min}}\#\left( 6 \right) \end{aligned}$$

where *X_n_* is the normalized value, *x* is the original data value, *x_min_* is the minimum value in the dataset and *x_max_* is the maximum value in the dataset. The results of multifactor parameterization for different structures are shown in **Table** S2.

The normalized parameter factors are averaged for real surface area and mass transfer capacity, then weighted scores are assigned to both. For the comprehensive score *S*:

$$\begin{aligned} S=\alpha_{i}\frac{\sum_{i=1}^{n} x_{i}}{n}+(1-\alpha_{i})\frac{\sum_{j=1}^{m} y_{j}}{m}\#\left( 7 \right) \end{aligned}$$

where *α_i_* is the weight of the real surface area, *x_i_* is the standardized score of the real surface area factors, *y_j_* is the standardized score of the mass transfer capacity factors.

**The influence of intrinsic surface chemistry, surface roughness, and mesoscale geometry on contact angle**

All samples were fabricated from the same batch of pure Ni by micro‑LPBF, underwent identical machining and post‑processing, and were subjected to a standardized 5‑min ultrasonic cleaning and drying protocol. All measurements were performed in the inert, as‑prepared state. Consequently, the surface chemistry is consistent across samples. We also quantified the average surface roughness of each architecture and provide the results in Table 1 and Table S1, showing that surfaces produced by micro‑LPBF exhibit very similar roughness; thus, surface roughness is not the primary factor governing the contact angles. Instead, meso‑scale geometry dictates the wetting response through curvature, specific surface area, and pore/neck features. Curved substrates modify the three‑phase contact‑line geometry and the matching of interfacial curvature; more continuous curvature as in the G and D structures, reduces the density of pinning sites, yielding smaller apparent contact angles at the same intrinsic contact angle. Increasing meso‑scale specific surface area generally enhances contact‑angle hysteresis; when the higher area arises from more tortuous pathways and numerous sharp edges (e.g., Koch structure), the apparent contact angle increases accordingly. Narrower necks and a larger cavity‑to‑throat ratio such as P structure, raise the depinning threshold of the contact line, enlarge the separation between advancing and receding angles, and thereby increase the apparent contact angle. Simultaneously, we tested the contact angle of bubbles in water and established a complementary relationship with the contact angle of water droplets in air, thereby mitigating the influence of chemical or roughness variations and attributing the discrepancy in contact angle to mesoscale geometry. Therefore, under comparable surface chemistry and roughness, differences in meso‑scale geometry are the dominant cause of the contact‑angle variations observed among the TPMS architectures.

4. **The correlation between static contact angle measurement and dynamic bubble separation behavior in operating electrolytic cells**

The adhesion force of bubbles on solid surfaces is controlled by three-phase lines, and whether bubbles generated by electrolysis detach depends on the competition between buoyancy and adhesion force. The adhesion force is F_adh_ ≈ γπD(1+cosθ_b_), and the buoyancy force is F_b_ ≈ ΔρgV. Detachment occurs when F_b_ ≥ F_adh_. The only factor controlled by surface wettability is 1+cosθ_b_. It monotonically increases as θ_b_ decreases, meaning that the adhesion of more hydrophobic structures is stronger, leading to larger and slower detachment of bubbles. Therefore, smaller contact angles or curvature will reduce adhesion and promote earlier detachment, so the low contact angle and small curvature of the G structure can make the dynamic detachment of bubbles faster. The contact angle of different structures we tested in water can reflect the ability of the structure to repel bubbles underwater and tends to cause bubbles to detach faster. The contact angle of bubbles in water and the dynamics of bubble separation have been thoroughly investigated. Li et al. ^[S1]^ developed a superhydrophobic electrode exhibiting an underwater contact angle of 180°, demonstrating exceptional dynamic bubble separation capabilities and rapid bubble diffusion times (~ 300 ms). Hu et al. ^[S2]^ systematically quantified the surface microstructure, contact angle, and oxygen bubble detachment threshold at varying current densities, revealing that larger underwater bubble contact angles correlate with earlier bubble detachment and reduced electrode surface coverage.

5. **Multi factor analysis of accelerated degradation of AEMWE stability.**

(i)	Continuous mechanical/chemical stress caused by flowing alkaline electrolyte

Undoubtedly, the impact of this on the degradation of electrode performance is evident. Figure S7 (b) shows the SEM image after failure, where the flowing alkaline electrolyte dissolves surface particles and shear forces cause unstable surface bonding, resulting in mechanical and chemical stress wear, leading to severe surface corrosion. However, due to the stability of the micro LPBF printed electrode, although voltage attenuation occurs after corrosion, it will not lead to failure. Because even with multiple layers of corrosion, the inner layer is still made of the same material but lacks a high microscopic surface area for reaction. In stability testing, it only reflects a rise in voltage rather than sudden failure. So, the failure condition is the result of multiple influencing factors.

(ii)	Anion exchange membrane specific influence factor different from static three electrode testing

Compared with the three-electrode system, anion exchange membrane is one of the main differences between the two systems. During long-term reactions, the two-electron reduction process of oxygen generates strong oxidative free radicals such as hydroxyl radicals, leading to polymer chain breakage or cross-linking, accelerating material aging and performance degradation. During long-term operation, the membrane is subject to erosion from electrolyte flow, tension during installation, and internal pressure of the stack, resulting in creep, tearing, or fatigue fracture. Under the impact of high flow velocity, repeated stress cycles will reduce its mechanical strength, which is the main factor causing the failure of the membrane mechanism. The recommended operating temperature for FAB-PK-130 is usually between 15-40 ℃, while the experimental temperature is 50 ℃, and further temperature increases during the reaction process, which accelerates the aging degree of the membrane. The attenuation of the membrane is one of the main reasons for the stability degradation of the electrolytic cell and is a key factor that distinguishes it from the long-term stability of the three-electrode system.

(iii)	Continuous bubble coverage and micro dry/wet cycling caused by two-phase flow, resulting in roughening and dehumidification of catalyst domains

In multiple cycles of bubble generation and separation, the surface of the cathode gas diffusion layer undergoes multiple wet dry cycles. When drying due to bubble coverage, the volume of the catalyst layer shrinks; The volume expands when the reactants are moist after supply. This tiny volume cycle generates alternating stress inside the material, and over time, the binding force between the catalyst nanoparticles and the carrier decreases, causing the catalyst attached to the carbon paper to become rough or even peel off. The catalytic effect of the final cathode is significantly reduced, which is one of the reasons for the stability failure.

**Table S1** Sample names, basic structure, overall size, unit cell dimensions and finished wall thicknesses of different 0.5 mm unit cell structures.

| Sample Name | Structure Function  [A=2πa/L, A=X, Y, Z] | Relative Density [%] | Wall Thickness [μm] | Average  Cross-sectional  Length [mm] | Surface Roughness Ra [μm] |
| --- | --- | --- | --- | --- | --- |
| 2D | $\phi_{D}\left( x,y,z \right)=\sin\left( 2X \right)\sin\left( 2Y \right)\sin\left( 2Z \right)+\sin\left( 2X \right)\cos\left( 2Y \right)\cos\left( 2Z \right)+\cos\left( 2X \right)\sin\left( 2Y \right)\cos\left( 2Z \right)+\cos\left( 2X \right)\cos\left( 2Y \right)sin(2Z)$ | 53.83 | 42.81 | 2374.88 | 1.64 ± 0.024 |
| 2G | $\phi_{G}\left( x,y,z \right)=\cos\left( 2X \right)\sin\left( 2Y \right)+\cos\left( 2Y \right)\sin\left( 2Z \right)+cos(2Z)sin(2X)$ | 43.26 | 46.76 | 1935.73 | 1.88 ± 0.047 |
| 2P | $\phi_{P}\left( x,y,z \right)=\cos\left( 2X \right)+\cos\left( 2Y \right)+\cos\left( 2Z \right)$ | 32.93 | 45.03 | 1454.68 | 1.76 ± 0.052 |
| 2Koch | $\phi_{Koch}\left( x,y,z \right)=\cos\left( 4X \right)\sin\left( 2Y \right)\cos\left( 2Z \right)+\cos\left( 2X \right)\cos\left( 4Y \right)\sin\left( 2Z \right)+\sin\left( 2X \right)\cos\left( 2Y \right)\cos\left( 4Z \right)$ | 67.20 | 43.25 | 3293.79 | 1.71 ± 0.034 |
|  |  |  |  |  | |

**Table S2** Multifactor parameterization results for different structures

| Sample Structure | Theoretical Surface Area | Relative Density | Contact Angle | Gas Flow Rate | Pressure Drop |
| --- | --- | --- | --- | --- | --- |
| G | 0.4032 | 0.30163 | 1 | 1 | 1 |
| D | 0.69268 | 0.61027 | 0.52062 | 0.61917 | 0.61271 |
| Koch | 1 | 1 | 0.23857 | 0 | 0 |
| P | 0 | 0 | 0 | 0.40681 | 0.80333 |

**Figure. S1** Macroscopic and corresponding microscopic schematic diagrams of 0.5 mm unit cell lattice structures with low-continuity printing defects highlighted in the graphs.

**Figure. S2** Microscopic morphology of 3D printed structure surface at different scales.

**Figure. S3** (a-e) CV curves measured in the non-faradaic region in 1 M KOH at scan rates of 10-50 mV s-1. (f) Calculation of double-layer capacitance (g) The LSV curve normalized by ECSA under the three-electrode system (h) The LSV curve normalized by ECSA under the AEMWE system with different structures.

**Figure. S4** Comparison of LSV curves of six G-structure specimens.

**Figure. S5** a) LSV diagrams of different structures under projected area. b) LSV diagrams of different structures under cross-sectional area. c) Tafel plots of different structures. d) EIS diagrams of different structures in three electrode system for 0.5 mm unit cell lattice structures.

**Figure. S6** Comparison of overpotential at 250 mA cm^-2^ of 0.5 mm unit cell structures.

**Figure. S7** SEM image of a) the three-electrode system after the stability test failure. b) the AEM electrolysis cell after the stability test failure.

**Figure. S8** The CV scan image of the G structure is used to determine the position of the oxidation-reduction peak.

**Figure. S9** Raman spectra before and after stability testing in two systems.

**Figure. S10** LSV curves and pH changes before and after stability testing in two systems.

**Figure. S11** Oxygen flow rate in the three-electrode system.

**Figure. S12** a) LSV diagrams of different structures under projected area. b) LSV diagrams of different structures under cross-sectional area. c) Tafel diagrams of 0.5 mm unit cell structures in AEM electrolytic cell.

**Figure. S13** a) Snapshots of contact angle for different 0.5 mm unit cell structures at 0 s, 0.1 s, and 0.2 s droplet contact times. b) Contact angle values for different structures.

**Figure. S14** Contact angles of underwater bubbles with different structures, and a comparison between it and those of the liquid in air at 0.2 second.

**Figure. S15** Corresponding outlet flow velocity for simulated data and experimental data.

**Figure. S16** Faraday efficiency and oxygen flow rate of different structures in AEMWE system.

**Figure. S17** (a) LSV curves normalized to projected area, (b) LSV curves normalized to ECSA, (c) Tafel slopes for three commercial PTLs.

**Figure. S18** a) Bubble streamlines in a homogeneous two-phase flow. b) Liquid streamlines of a homogeneous two-phase flow. c) The volume fraction of the dispersed phase in xz-plane cross-section. d) Top view of liquid streamlines. e) The volume fraction of the dispersed phase at the gas interface.

**References**

[S1]	L. Li, P. C. M. Laan, X. Yan, X. Cao, M. J. Mekkering, K. Zhao, L. Ke, X. Jiang, X. Wu, L. Li, L. Xue, Z. Wang, G. Rothenberg, N. Yan, *Advanced Science* **2023**, *10*, 2206180.

[S2]	J. Hu, C. Guo, J. Fan, M. A. Basit, Y. Shi, Q. Bao, L. Jiang, C. Yan, *Electrochimica Acta* **2025**, *541*, 147115.
